# Supplementary material for: Microglia-Derived Spp1 Promotes Pathological Retinal Neovascularization via Activating Endothelial Kit/Akt/mTOR Signaling
Source: J Pers Med. 2023 Jan 11;13(1):146. doi: 10.3390/jpm13010146 (PMC9866717; doi:10.3390/jpm13010146)
Supplement: Supplementary file 1 [file jpm-13-00146-s001.zip › jpm-2073583-supplementary.pdf]

Supplementary Materials

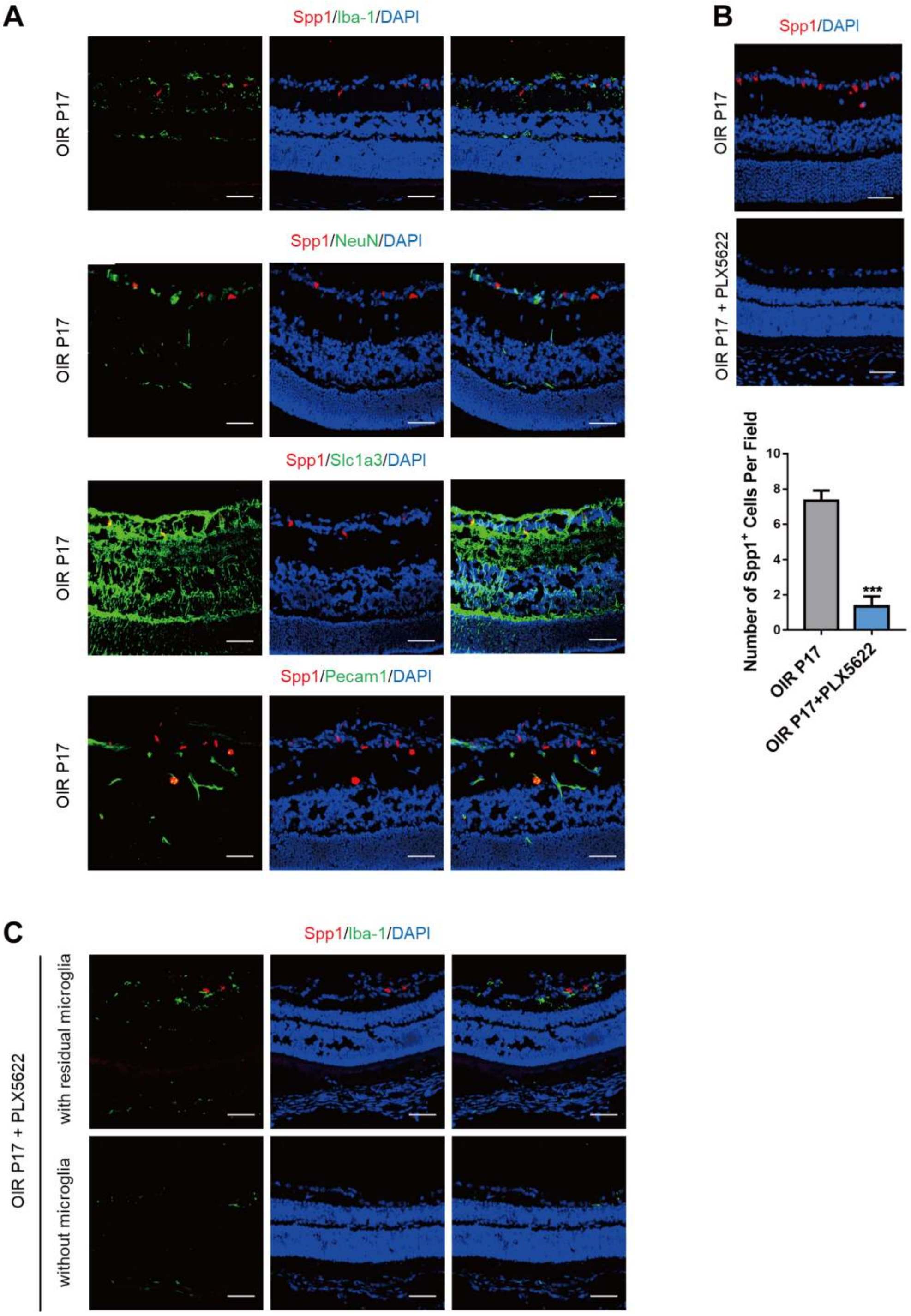

Figure S1. Spp1 was mainly secreted from Microglia. (A) Representative immunofluorescence images of eye balls from OIR P17 stained with Spp1 (red), Iba-1, NeuN, Slc1a3, Pecam1 (green, respectively) and DAPI (blue). Scale bars: 50 μm. (B) Representative immunofluorescence images of eye balls of from OIR P17 or OIR P17 treated with PLX5622 stained with Spp1 (red) and DAPI (blue). Numbers of Spp1$^+$ cells were quantified per field. (mean ± SD; n = 3/group; ***$P < 0.001$, unpaired Student’s t-test). Scale bars: 50 μm. (C) Representative immunofluorescence images of eye balls from OIR P17 treated with PLX5622 stained with Spp1 (red), Iba-1 (green) and DAPI (blue). Areas with or without residual microglia were showed. Scale bars: 50 μm.

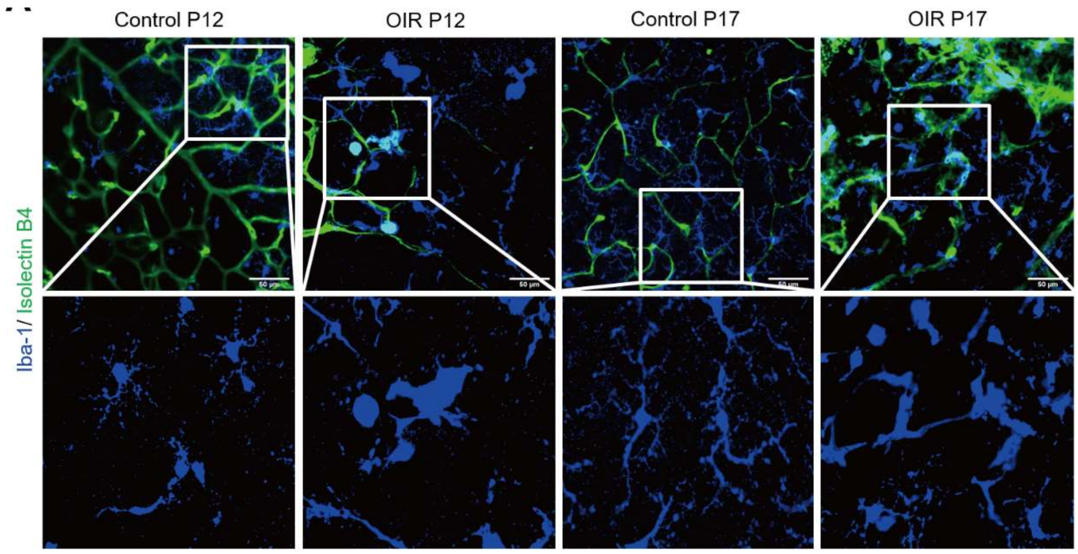

Figure S2. Microglia in OIR presents an active feature. Representative immunofluorescence images of whole retinal flat-mounts in Control P12, OIR P12, Control P17 and OIR P17 stained with IB4 (green) and Iba-1 (blue). In normal retinas (Control P12 and Control P17), microglia adopted a resting state featured with small cell bodies with thin and long processes. In contrast, activated microglia, characterized by enlarged cell somas with thick and short lamellipodia, were found in avascular area and around NV tufts in OIR retinas (OIR P12 and OIR P17).

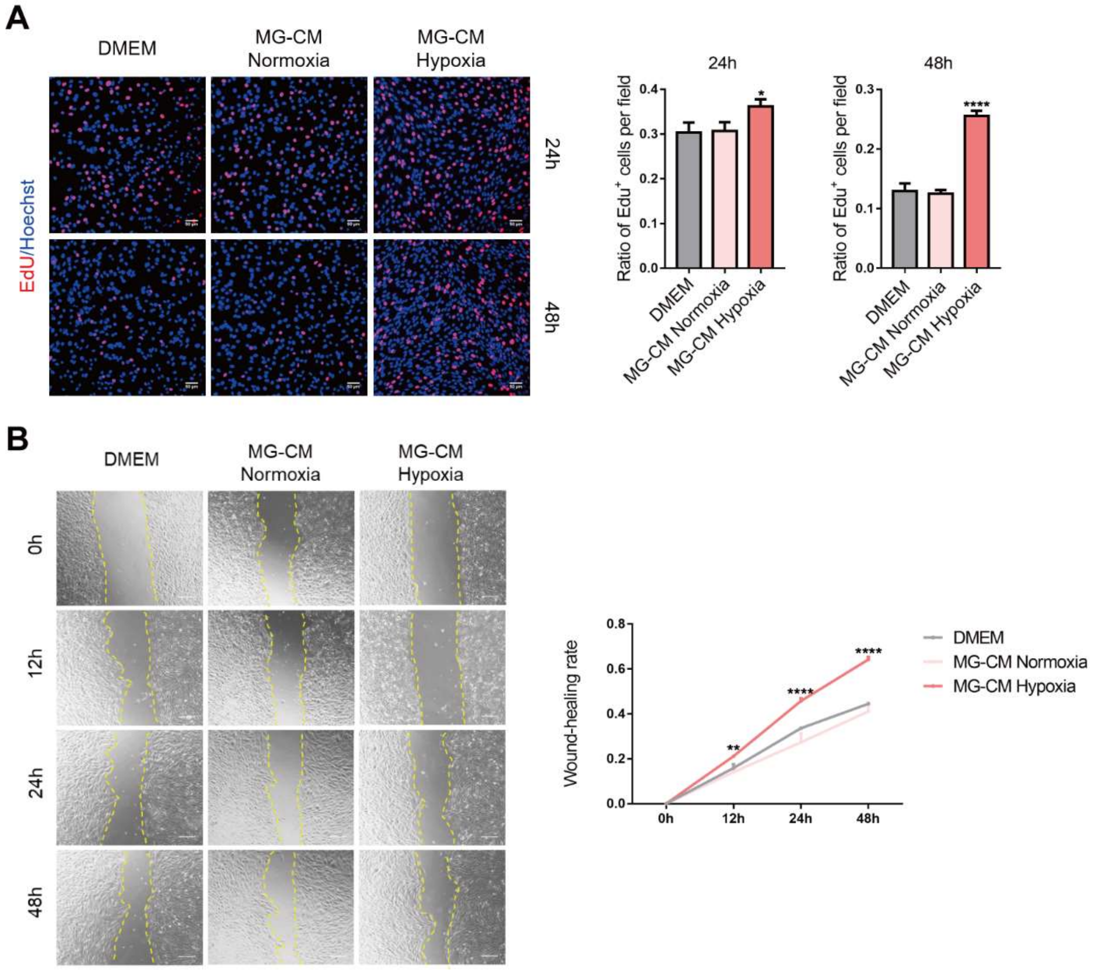

Figure S3. CM from hypoxia-cultured microglia promotes endothelial proliferation and migration in vitro. (A) Results from EdU incorporation assay showing the proliferation capacity of bEnd.3 treated with DMEM, MG-CM from normoxia- or hypoxia-cultured BV2 (mean ± SD; n = 3/group , three visual fields were taken from one well to calculate the average value; one-way ANOVA). Scale bars: 50 μm. (B) Results from Wound healing assay showing the migration rate of bEnd.3 treated with DMEM, MG-CM from normoxia- or hypoxia-cultured BV2 (mean ± SD; n = 3/group, three visual fields were taken from one well to calculate the average value; one-way ANOVA). Scale bars: 200 μm. *$P < 0.05$, **$P < 0.01$, ****$P < 0.0001$.
